# Effectiveness, cost-utility and physiological underpinnings of the FIBROWALK multicomponent therapy in online and outdoor format in individuals with fibromyalgia: Study protocol of a randomized, controlled trial (On&Out study)

**DOI:** 10.3389/fphys.2022.1046613

**Published:** 2022-11-10

**Authors:** Mayte Serrat, Sònia Ferrés, William Auer, Míriam Almirall, Enrique Lluch, Francesco D’Amico, Michael Maes, Sonia Lorente, Jaime Navarrete, Jesús Montero-Marín, Randy Neblett, Jo Nijs, Xavier Borràs, Juan V. Luciano, Albert Feliu-Soler

**Affiliations:** ^1^ Unitat d’Expertesa en Síndromes de Sensibilització Central, Servei de Reumatologia, Vall d’Hebron Hospital Universitari, Vall d’Hebron Barcelona Hospital Campus, Barcelona, Spain; ^2^ Escoles Universitàries Gimbernat, Autonomous University of Barcelona, Barcelona, Spain; ^3^ Department of Basic, Developmental and Educational Psychology, Faculty of Psychology, Autonomous University of Barcelona, Barcelona, Spain; ^4^ Department of Physiotherapy, Faculty of Physiotherapy, University of Valencia, Valencia, Spain; ^5^ Physiotherapy in Motion, Multi-Specialty Research Group (PTinMOTION), Department of Physiotherapy, University of Valencia, Valencia, Spain; ^6^ Pain in Motion International Research Group, Brussels, Belgium; ^7^ Care Policy and Evaluation Centre (CPEC), London School of Economics and Political Science (LSE), London, United Kingdom; ^8^ Department of Psychiatry, Faculty of Medicine, Chulalongkorn University, Bangkok, Thailand; ^9^ Department of Psychobiology and Methodology of Health Science, Autonomous University of Barcelona, Barcelona, Spain; ^10^ Pediatric Area, PNP, Hospital de Terrassa, Consorci Sanitari de Terrassa, Terrassa, Spain; ^11^ Psychological Research in Fibromyalgia and Chronic Pain (AGORA Research Group), Parc Sanitari Sant Joan de Déu, Barcelona, Spain; ^12^ Warneford Hospital, Department of Psychiatry, University of Oxford, Oxford, United Kingdom; ^13^ PRIDE Research Foundation, Dallas, TX, United States; ^14^ Pain in Motion Research Group (PAIN), Department of Physiotherapy, Human Physiology, and Anatomy, Faculty of Physical Education & Physiotherapy, Vrije Universiteit Brussel, Brussels, Belgium; ^15^ Department of Health and Rehabilitation, Unit of Physiotherapy, Institute of Neuroscience and Physiology, Sahlgrenska Academy, University of Gothenburg, Gothenburg, Sweden; ^16^ Chronic Pain Rehabilitation, Department of Physical Medicine and Physiotherapy, University Hospital Brussels, Bierbeek, Belgium; ^17^ Department of Clinical and Health Psychology, Faculty of Psychology, Autonomous University of Barcelona, Barcelona, Spain

**Keywords:** fibromyalgia, online, outdoor, pain neuroscience education, effectiveness, cost-utility, cytokines, BDNF

## Abstract

**Introduction:** The On&Out study is aimed at assessing the effectiveness, cost-utility and physiological underpinnings of the FIBROWALK multicomponent intervention conducted in two different settings: online (FIBRO-On) or outdoors (FIBRO-Out). Both interventions have proved to be efficacious in the short-term but there is no study assessing their comparative effectiveness nor their long-term effects. For the first time, this study will also evaluate the cost-utility (6-month time-horizon) and the effects on immune-inflammatory biomarkers and Brain-Derived Neurotrophic Factor (BDNF) levels of both interventions. The objectives of this 6-month, randomized, controlled trial (RCT) are 1) to examine the effectiveness and cost-utility of adding FIBRO-On or FIBRO-Out to Treatment-As-Usual (TAU) for individuals with fibromyalgia (FM); 2) to identify pre–post differences in blood biomarker levels in the three study arms and 3) to analyze the role of process variables as mediators of 6-month follow-up clinical outcomes.

**Methods and analysis:** Participants will be 225 individuals with FM recruited at Vall d’Hebron University Hospital (Barcelona, Spain), randomly allocated to one of the three study arms: TAU vs. TAU + FIBRO-On vs. TAU + FIBRO-Out. A comprehensive assessment to collect functional impairment, pain, fatigue, depressive and anxiety symptoms, perceived stress, central sensitization, physical function, sleep quality, perceived cognitive dysfunction, kinesiophobia, pain catastrophizing, psychological inflexibility in pain and pain knowledge will be conducted pre-intervention, at 6 weeks, post-intervention (12 weeks), and at 6-month follow-up. Changes in immune-inflammatory biomarkers [i.e., IL-6, CXCL8, IL-17A, IL-4, IL-10, and high-sensitivity C-reactive protein (hs-CRP)] and Brain-Derived Neurotrophic Factor will be evaluated in 40 participants in each treatment arm (total n = 120) at pre- and post-treatment. Quality of life and direct and indirect costs will be evaluated at baseline and at 6-month follow-up. Linear mixed-effects regression models using restricted maximum likelihood, mediational models and a full economic evaluation applying bootstrapping techniques, acceptability curves and sensitivity analyses will be computed.

**Ethics and dissemination:** This study has been approved by the Ethics Committee of the Vall d’Hebron Institute of Research. The results will be actively disseminated through peer-reviewed journals, conference presentations, social media and various community engagement activities. Trial registration number NCT05377567 (clinicaltrials.gov).

## 1 Introduction

Fibromyalgia (FM) is a highly prevalent syndrome (2.7% worldwide, 2.5% in Europe and 2.4% in Spain) (Queiroz, 2013) which mainly affects women, around 40–50 years of age (Häuser et al., 2015). FM is characterized by the presence of chronic widespread musculoskeletal pain, fatigue and sleep problems (Häuser et al., 2015) and, very often, it is also accompanied by anxiety (13%–63.8%) and depressive disorders (20%–80%) (Fietta et al., 2007). Remarkably, FM is the chronic pain condition with the highest rate of unemployment, sick leave, disability claims and absenteeism (Leadley et al., 2012) and 23%–66% of individuals with this syndrome are forced to leave work due to its aftermaths (Assefi et al., 2003; Gerdle et al., 2008). Furthermore, individuals with FM are usually considered “hyper-frequent users'' of health services with 6% of adult patients attending primary care and 10%–20% attending rheumatology services having a diagnosis of FM (Gerdle et al., 2008; Häuser et al., 2015). Regarding this latter point, FM is the medical condition presenting higher costs, with direct costs (i.e., medication, visits to healthcare professionals, medical tests, emergency room visits and hospital admissions) being up to three times higher than the observed in other chronic pain conditions and similar sociodemographic characteristics (Berger et al., 2007; Leadley et al., 2012; Wylezinski et al., 2019).

### 1.1 Recommended treatments for FM and the emergence of online and outdoors therapeutic approaches

Up to date, the available treatments for FM are not curative and their clinical efficacy is generally rather low (Häuser et al., 2015). In relation to pharmacological treatments, the European League Against Rheumatism (EULAR) recommends reserving pharmacological treatment only for those cases with severe pain and for sleep disturbances (Häuser et al., 2015; Macfarlane et al., 2017). In line with this, non-pharmacological strategies appear to promote broader clinical effects and show slightly larger effect sizes compared to pharmacological treatments (Nüesch et al., 2013, Perrot et al., 2014). Non-pharmacological strategies include interventions such as pain education, cognitive behavioral therapy (CBT), mindfulness, therapeutic physical exercise, among others, which aim primarily at alleviating symptoms and improving patients’ quality of life (Macfarlane et al., 2017). More specifically, Pain Neuroscience Education (PNE) (Van Oosterwijck et al., 2013; Nijs et al., 2014; Amer-Cuenca et al., 2020) is aimed at changing patients’ pain beliefs, emphasizing how overprotective behaviors can accentuate pain experience (Moseley and Butler, 2015). PNE has been found to be effective for reducing pain disability, catastrophizing, avoidance behaviors and physical inactivity in patients with FM (Malfliet et al., 2017). Concurrently, there is high agreement among clinical guidelines that CBT and therapeutic physical exercise should constitute the main therapeutic elements when treating FM (Jones et al., 2006; Bernardy et al., 2010; Sosa-Reina et al., 2017; Bernardy et al., 2018); furthermore, combining both interventions has been shown to be particularly effective (Witek-Janusek et al., 2008; Black et al., 2013). There is also mounting evidence that mindfulness training can be an efficacious and cost-effective approach for improving quality of life, functional impairment, anxiety, depression, and other symptoms in patients with FM (Haugmark et al., 2019; Pérez-Aranda et al., 2019).

Mounting empirical evidence suggests that multicomponent approaches integrating at least therapeutic physical exercise and a psychological/educational intervention can be effective in individuals with FM (e.g., Van Wilgen et al., 2007; Castel et al., 2013; Thieme et al., 2017) and some authors propose that these multicomponent interventions should be the “gold standard” in FM (Rivera et al., 2006; Häuser et al., 2008; De Miquel et al., 2010; Macfarlane et al., 2017; Thieme et al., 2017). In this regard, a metanalysis exploring the efficacy of different pharmacological and non-pharmacological therapies on fibromyalgia symptoms (Papadopoulou et al., 2016) suggested that multidisciplinary treatments would be the most beneficial ones among non-pharmacological interventions for treating FM, since statistically significant improvements were found in all FM symptoms comprising OMERACT-10 response criteria (i.e., pain, sleep, function, fatigue, anxiety, depression, cognition). Furthermore, four recent RCTs published after the aforementioned metanalysis, provided additional evidence on the short-term efficacy (i.e., post-intervention) of a 3-month multicomponent intervention for FM (i.e., the FIBROWALK program) comprising CBT, mindfulness training, therapeutic physical exercise and PNE (Serrat et al., 2020; Serrat et al., 2021a; Serrat et al., 2021b; Serrat et al., 2022b). Interestingly, the FIBROWALK program showed to be clinically efficacious when tested in hospital, outdoors and also in online settings compared to Treatment-As-Usual (TAU) alone and provided clinically relevant improvements in functionality, pain, kinesiophobia, physical function, fatigue, and anxiety and depressive symptomatology (Serrat et al., 2020; Serrat et al., 2021a; Serrat et al., 2021b; Serrat et al., 2022b).

The COVID-19 pandemic has enforced the emergence of new therapeutic approaches alternative to face-to-face treatment since mobility restrictions and infection risk have generally limited this classical format of therapy. In this regard, there is a growing body of evidence on the efficacy of alternative forms of therapy such as online (Schwamm et al., 2020; Weiling et al., 2020; Serrat et al., 2021a; White et al., 2022) and outdoor (Buckley et al., 2018; Stanhope et al., 2020) formats in chronic pain populations including FM.

Beyond pandemics, teletherapy approaches such as online FIBROWALK (Serrat et al., 2021a) are highly promising when compared to traditional face-to-face when dealing with specific logistic barriers such as time, travel or access difficulties in rural areas, or health barriers such as patients’ fatigue, among other symptoms which may interfere with treatment adherence. Online programs which are highly scalable and accessible per definition may also help in decongest healthcare services (which are particularly overstretched worldwide since the COVID-19 pandemic), significantly reduce healthcare staff costs, and help increase widespread access to evidence-based treatment for FM (Assefi et al., 2003; Friedman et al., 2012; Moman et al., 2019). In this regard, in a recent systematic review on the efficacy of online psychological interventions for people with chronic health conditions, online treatments were found to be efficacious for improving depressive symptomatology and distress in people with FM, supporting the usefulness of these low cost, easily accessible, and highly scalable therapeutic approaches for treating this syndrome (White et al., 2020). Furthermore, an important aspect when implementing treatments, especially for chronic conditions, is to promote self-management and online interventions may facilitate this aspect (Leadley et al., 2012). Simultaneously, nature-based therapeutic approaches such as the outdoor FIBROWALK (Serrat et al., 2020) have shown to be useful in improving mental health in different clinical conditions (Trøstrup et al., 2019), including chronic pain and FM (López-Pousa et al., 2015; Serrat et al., 2020; Stanhope et al., 2020). In this sense, it is interesting that therapeutic programs conducted outdoors have been shown to be effective in improving mental health, pain and general health, and it is suggested that exposure to the natural environment (both in urban green areas and in nature) may produce additive positive effects at both affective (positive and negative emotions and stress) and cognitive (attention, memory, motivation, *etc.*) levels thus enhancing the beneficial effects of the program (Buckley et al., 2018). In this regard, when compared to therapeutic exercise conducted indoors, exercising in natural settings was associated with greater feelings of revitalization and positive engagement, reductions in tension, confusion, anger, and depressive symptomatology, and increased energy (Thompson Coon et al., 2011). Furthermore, there is evidence that exercising outdoors (vs. indoors) may also promote directed attention and social interactions, which may positively influence future intention of maintaining an exercise routine (Rogerson et al., 2016). Regarding the possible effects of the intervention format of the FIBROWALK intervention was found to be beneficial in hospital, outdoors and virtual settings at short term with apparently larger effect sizes in the outdoor format when compared to indoor hospital or online settings (Serrat et al., 2020; Serrat et al., 2021a; Serrat et al., 2021b; Serrat et al., 2022b). However, no clinical trial with a parallel design has yet been conducted to evaluate potential differences in the short- and the long-term efficacy between these different formats or in therapeutic adherence. The present study aims to address this important knowledge gap.

Although recommended, there is a striking lack of empirical evidence regarding the cost-effectiveness of multicomponent interventions for FM, including FIBROWALK. Bearing in mind that costs are of crucial relevance for policy-makers, who usually consider as first-choice treatment those therapeutic approaches with the lowest associated cost per quality-adjusted life-year (QALY), economic evaluations of multicomponent interventions in FM should be performed to ensure (if cost-effective) their implementation in healthcare systems.

### 1.2 Immune-inflammatory status and BDNF levels in individuals with FM

There are several potential physiological factors which have been proposed underpinning the FM symptomatology. In this regard, the central nervous system (CNS) seems to have a leading role (Häuser et al., 2015), involving altered function in pain neural pathways (Sluka and Clauw, 2016; Sawaddiruk et al., 2017). At the same time, there is evidence suggesting an imbalance between pro- and anti-inflammatory cytokine levels at both the peripheral and central levels in individuals with FM contributing to a chronic state of low-grade inflammation (Rodriguez-Pintó et al., 2014; Bäckryd et al., 2017; Albrecht et al., 2019; Andrés-Rodríguez et al., 2019). This proinflammatory state may be a key contributor to impaired pain processing as could lead to the sensitization of peripheral nerves and to central sensitization (Rodriguez-Pintó et al., 2014; Littlejohn, 2015), possibly through activation of glial cells (Nijs et al., 2017).

A recent meta-analysis concluded that patients with FM may present increased levels of blood IL-6, IL-4 and IL-17A compared to healthy controls (Andrés-Rodríguez et al., 2020). Coherently, increased levels in other markers indicative of an inflammatory state [e.g., high-sensitivity C-reactive protein (hs-CRP)] were also reported (Andrés-Rodríguez et al., 2020). Some of these proinflammatory cytokines (e.g., IL-6) are known to facilitate sensitization of peripheral nerves to nociceptive stimuli and to increase the activity of the hypothalamic-pituitary-adrenal axis, as well as prostaglandin and substance P synthesis, thereby reducing the pain threshold (Zhou et al., 2016). On the other hand, at a central level, pro-inflammatory cytokines along with other inflammatory-related by-products are known to alter the synthesis, reuptake and release of different neurotransmitters in the CNS (e.g., serotonin, noradrenaline, dopamine, glutamate. These neurotransmitters are involved in both pain perception and the regulation of diverse affective, cognitive and motivational processes, contributing to the neuroplastic changes observed in FM, abnormal pain experience and to other FM symptoms, such as fatigue, sleep disturbances, cognitive impairment, and affective problems (Miller et al., 2013; Rodriguez-Pintó et al., 2014; Andrés-Rodríguez et al., 2020).

Several studies have suggested that FM and other central sensitivity syndromes present not only with chronic low-grade inflammation but also with abnormalities in biomarkers related to neuronal plasticity, such as BDNF (Deitos et al., 2015). The BDNF is a multifunctional neurotrophin with numerous functions in the brain (Jensen et al., 2012; Cagnie et al., 2014), including a driving force behind (maladaptive) neuroplasticity and central sensitization (Nijs et al., 2015), and it is believed to be specifically associated with the occurrence and/or progression of mnemonic symptoms associated with a variety of conditions characterized by cognitive impairment (Napadow et al., 2012). BDNF is known to play a key role in a variety of neuroplasticity processes, including pain modulation, pain transduction, nociception, and hyperalgesia (Nugraha et al., 2012), all of which are known to be altered in FM. In this regard, plasma levels of BDNF have also been found to be augmented in individuals with FM (Haas et al., 2010; Polli et al., 2020) and DNA methylation in exon nine appears to be driving the higher BDNF protein levels in patients with FM (Polli et al., 2020); however, we have to bear in mind that associations between BDNF levels and patients’ clinical complaints have not been found always consistent (Zanette et al., 2014; Jha and Trivedi, 2018) and that some studies have found no differences between FM patients and healthy controls on BDNF levels (Ranzolin et al., 2016). Furthermore, there are reciprocal associations between BDNF and immune-inflammatory pathways, which may explain the involvement of BDNF in the immune pathophysiology of mood disorders (Perrot and Russell, 2014), with BDNF acting as a conserved regulatory process that protects against the detrimental effects of immune activation including neurotoxicity (Mehterov et al., 2022).

Some of these biomarkers may also influence treatment response in individuals with chronic pain conditions including FM. Related to this, higher pretreatment levels of IL-6 and TNF-α have been found to be associated with lesser improvements in pain intensity and other patient-reported outcomes after cognitive behavioral interventions administered in individuals with chronic pain (Lasselin et al., 2016). Furthermore, higher baseline levels of CXCL8 were also found to attenuate clinical benefits (i.e., in pain, fatigue, stiffness and quality of sleep) after an 8-week standardized mindfulness program in patients with FM (Andrés-Rodríguez et al., 2019).

### 1.3 Effects of non-pharmacological interventions on immune-inflammatory markers and BDNF in individuals with FM

In a systematic review of longitudinal studies in FM, multicomponent programs, physical exercise and dietary modification were found to have effects on inflammatory markers in FM patients (Sanada et al., 2015). In this regard, reductions in levels of the pro-inflammatory cytokines IL-6 and CXCL8 were observed in FM patients following these interventions, suggesting a potential anti-inflammatory effect. Similarly, in a recent study (Andrés-Rodríguez et al., 2019) evaluating the effects of mindfulness training on immune-inflammatory markers, it was also reported that levels of the anti-inflammatory cytokine IL-10 remained higher in patients assigned to mindfulness than those allocated to the TAU arm. In another study assessing the effects of an 8-week compassion-based intervention (i.e., Attachment-Based Compassion Therapy, ABCT) for FM, a global anti-inflammatory effect along with reductions in BDNF levels in individuals with FM when compared to an active control condition (i.e., relaxation training) were also reported (Montero-Marin et al., 2019). These changes in BDNF levels were significantly related and followed by ameliorations in FM functional impairment, showing significant indirect effects, which means that reductions in BDNF might function as a crucial treatment mediator. In this study, baseline BDNF levels were similar to those obtained in a previous study with patients suffering from central sensitivity syndromes with persistent somatic or visceral nociception in which, after treatment, these levels normalized, approaching to those observed in pain-free individuals (Deitos et al., 2015). Regarding the effects of psychosocial interventions (mainly psychotherapy and psychoeducation) on immune function in adults with different health conditions, in an extensive systematic review and meta-analysis (Shields et al., 2020), many different improvements in immune function (including levels of pro-inflammatory biomarkers) were robustly reported. Interestingly, these effects were more remarkable after the application of CBT and multicomponent programs. Importantly, there are no available studies assessing the effects of multicomponent interventions such as FIBROWALK in physiological markers in fibromyalgia aimed at understanding physiological mechanisms underpinning its efficacy.

The objectives of the On&Out study are 1) to examine the effectiveness and cost-utility of adding the FIBROWALK intervention in online or in outdoors format to TAU in the management of patients with FM for improving functional impairment (primary outcome) and pain, fatigue, depressive and anxiety symptoms, perceived stress, central sensitization, physical function, sleep quality and perceived cognitive dysfunction (secondary outcomes); 2) to identify pre–post differences in levels of different physiological variables (i.e., immune-inflammatory markers and BDNF levels) and correlate these changes with those observed at self-report measures and 3) evaluate the role of psychological process variables considered to be potential mediators of the interventions from a theoretical point of view (i.e., kinesiophobia, pain catastrophizing, psychological inflexibility in pain and pain knowledge) and the evaluated physiological variables as mediators of long-term clinical improvements. For the sake of personalized treatment in FM, the design of the present study will allow us to explore whether certain patient profiles or baseline psychobiological characteristics may help predict the short- and long-term clinical response to the specific evaluated treatments.

## 2 Materials and methods

### 2.1 Trial design

**FIGURE 1:**
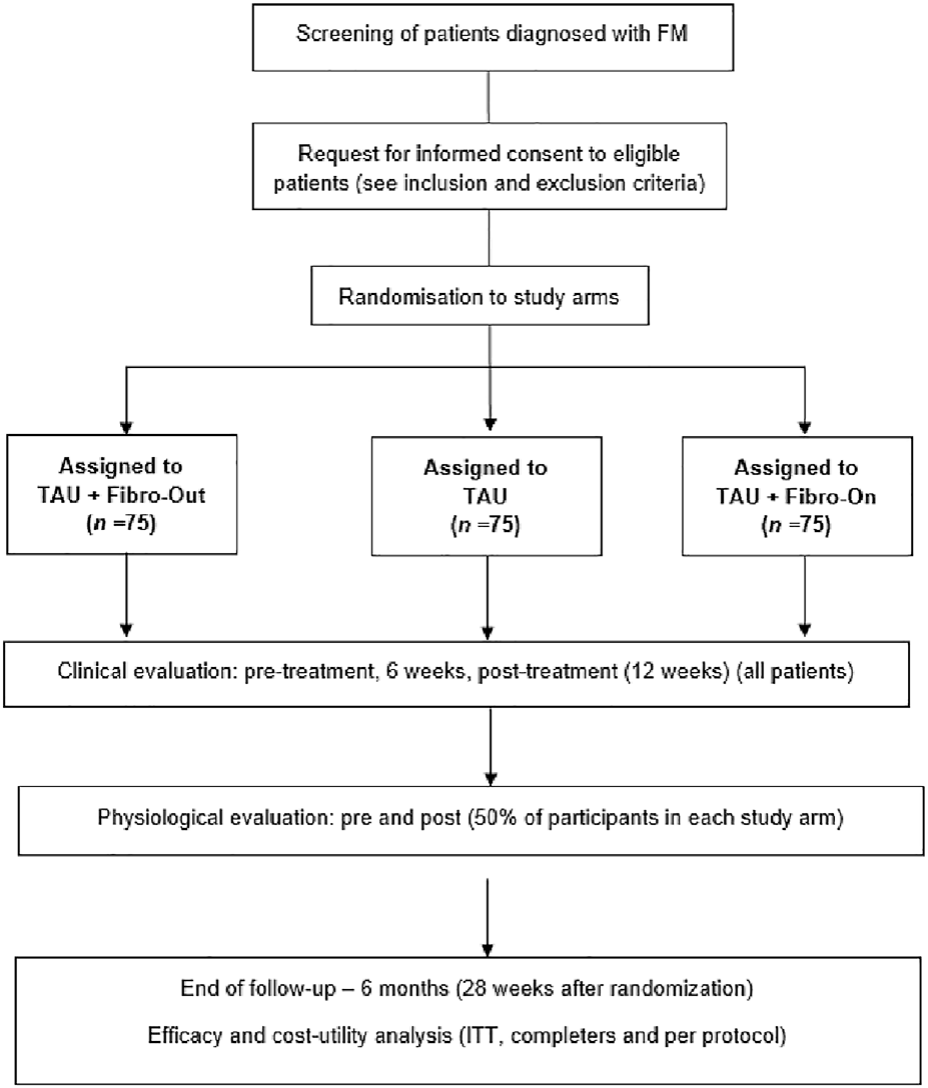
Flowchart of the On&Out study based on the Consolidated Standards of Reporting Trials guidelines. Fibro-Out, FIBROWALK Outdoors; Fibro-On, FIBROWALK Online; ITT, intention-to-treat; TAU, Treatment-As-Usual.

This RCT protocol was developed following the Standard Protocol Items: Recommendations for Interventional Trials (SPIRIT) (Chan et al., 2013) and was recorded in the ClinicalTrials.gov trial register in May 2022 (NCT05377567). We designed a 6-month, parallel group, randomized (using a computer-generated randomization list), single-blind, controlled trial (RCT) with three treatment arms. The effectiveness of the FIBROWALK program (i.e., multicomponent program based on therapeutic exercise, PNE, CBT, and mindfulness training) in FIBRO-On (online) and in FIBRO-Out (outdoors) formats adjuvant to TAU, compared to TAU alone in patients with FM recruited from the Central Sensitivity Syndromes Specialized Unit from the HUVH, will be evaluated. The Consolidated Standards of Reporting Trials 2010 (CONSORT) (Schulz et al., 2010) and the Consolidated Health Economic Evaluation Reporting Standards (CHEERS) (Husereau et al., 2013) will be followed. The expected flowchart of participants in the study is displayed in Figure 1.

### 2.2 Recruitment strategy

Potential participants are individuals with FM diagnosis (ACR 2010/2011 criteria) made by a rheumatologist and seeking health services currently or in the last year at the Central Sensitivity Syndromes Specialized Unit at HUVH.

### 2.3 Sample size calculation

Two-hundred and twenty-five individuals who meet the selection criteria for the study will be recruited. The estimated sample size needed is n = 63 participants per condition considering a moderate effect size (Cohen’s d = 0.5) for the difference between the active arms at post-intervention compared to the control group for the primary variable (i.e., FIQR score) with an alpha = 0.05 and 1-b power = 0.80. With an expected dropout rate of 20%–25%, the final sample size is estimated at 75 individuals per arm. The effect size used for the sample size calculation corresponds to the average effect size for multicomponent interventions in FM vs. control group (Nüesch et al., 2013). Previous RCTs evaluating the FIBROWALK intervention indicated effect sizes between d = 0.4 (Serrat et al., 2021a) and d = 1.83 (Serrat et al., 2020). Using as a reference previous studies of the group evaluating the effect of different non-pharmacological treatments in FM patients on blood values of immuno-inflammatory markers (Andrés-Rodríguez et al., 2019; Montero-Marin et al., 2020), it is estimated that a sample size of 40 subjects per arm will be sufficient to be able to detect changes in the evaluated variables.

### 2.4 Eligibility criteria

All participants will meet the following general inclusion criteria: 1. Individuals between 18 and 65 years of age. 2. Diagnosis of FM according to the ACR 2010/2011 criteria. 3. Understanding written and spoken Spanish. The general exclusion criteria will be: 1. Psychological treatment received within the last year or current. 2. Comorbid presence of severe mental disorder (e.g., schizophrenia) or other terminal clinical conditions or scheduled treatments that may interrupt study follow-up. 3. Inability to complete the weekly sessions/modules of the program on a regular basis.

For the biomarkers substudy, usual contraindications for measuring blood immune-inflammatory markers will be taken into account to establish the following additional inclusion/exclusion criteria for the biomarkers substudy: only participants of female sex, with no autoimmune-type disease, no recent physical trauma, not being vaccinated during the last week, no symptoms suggestive of cold/infection on the day of blood collection, no needle phobia, not pregnant or breastfeeding, not using oral or local corticosteroids or any anti-cytokine biologic drugs or oral contraceptives will be included in the biomarkers substudy.

### 2.5 Procedure and randomization

The recruitment of participants will be carried out in two ways: 1) consecutive recruitment from consultation by the rheumatologists of the HUVH Central Sensitivity Syndromes Specialized Unit and 2) by screening a list of patients (with contact telephone number) corresponding to patients who contacted the Unit during the last 6 months. The clinicians involved in the project (i.e., MA and MS) will proceed to carry out the telephone screening and those who meet the selection criteria of the study will be invited to participate. After obtaining written informed consent, the instrument battery -including clinical and economic measures-described below will be administered (online assessment). Two research assistants will coordinate the study assessments to ensure an adequate understanding of all administered self-reported measures. Random assignment of participants to study arms will be executed after baseline assessments as recommended by the CONSORT guidelines (Schulz et al., 2010). Once written consent and baseline assessment have been completed, study participants will be given a unique personal code and randomized using randomization software (1:1:1 ratio). The computer-generated randomization will apply a permuted block design to ensure that the groups are balanced taking biomarkers substudy eligibility criteria (No=Not meeting criteria for biomarkers substudy; Yes = Meeting criteria) into account. The randomization list will be stored in an encrypted file on a password-protected computer in the clinical trials supervisor’s office to assure concealment of allocation. Participants will be informed of their group allocation by the administrative personnel, who will send a notification *via* email.

Patients who meet the selection criteria for the biomarkers substudy will be rescheduled 3–5 days after the first assessment to obtain blood samples (up to the required N of 40 subjects per arm). Fasting blood extractions will be performed in a pre-set time window (8a.m.–10a.m.) to reduce circadian variability in the levels of the biomarkers evaluated. In order to limit the effects of medication on the study variables, patients will be asked to refrain from taking any occasional anti-inflammatory drugs in the 72 h prior to blood sampling.

The research assistants, who will be blinded to randomization, will coordinate the three subsequent clinical assessments: at 6 weeks, at post-intervention and at 6 months after the baseline assessment (follow-up). The same procedure as in baseline will be followed for the collection of biological samples at post-intervention, respecting the pre-set time interval.

The study will be organized in two consecutive periods with six study groups (i.e., 2 TAU, 2 FIBRO-Out, 2 FIBRO-On) conducted in parallel in each period. All the groups will include 18–20 participants (i.e., FIBRO-On, FIBRO-Out, TAU), so the N = 75 is expected to be reached at the end of the study.

### 2.6 Interventions

#### 2.6.1 TAU

Although there is no *gold standard* treatment for FM, in the Spanish healthcare context, the TAU for FM is mainly pharmacological and is adjusted to the symptomatologic profile of each patient. It is usually also accompanied by guidelines for aerobic physical exercise adapted to the individual’s limitations. Participants assigned to this treatment arm will be offered to participate in the FIBRO-On or FIBRO-Out program (whichever is their choice) at the end of the study.

#### 2.6.2 FIBRO-On

This program is also based in the FIBROWALK program (Serrat et al., 2020) and is virtually identical to the FIBRO-Out program described below. The FIBRO-On program will also be administered as an adjuvant intervention to TAU. All contents of the FIBROWALK (explanations and guidelines for the practice of the components of PNE, physical exercise, CBT and mindfulness) will be explained by means of videos together with slide presentations. The contents in the videos will be taught by the first author (MS) who is both a physiotherapist with more than 17 years of experience and a health psychologist with more than 8 years of experience. Furthermore, MS was the creator of the FIBROWALK program. Although the virtual sessions are 60 min long, a general recommendation is to expend 120 min to perform all the exercises indicated in each specific video. Every week, links to the videos of the corresponding module will be sent by email so that patients can access them whenever possible during the week. To limit problems of access to the videos by patients with little technological experience, a highly-accessible and user-friendly platform will be used (e.g. Youtube). To verify that participants adhered to FIBRO-On program, participants will be asked to complete a brief weekly questionnaire (5–10 items) asking for very basic concepts explained in the videos (e.g., “Please, provide a short example of a catastrophic thought”). Participants who do not answer the weekly questionnaire or those informing of any issue interfering with the adherence to treatment protocol (e.g., not being able to do the homework, watching the videos, answering the questionnaire, *etc.*) will be contacted (*via* SMS and/or telephone calls) by the therapist in charge (MS) and some guidance will be facilitated. If necessary, individuals who were unable to view or answer the questionnaire in a specific week could request an extension of the date. There was no therapeutic interaction with the participants, but participants were invited to contact the therapist by email if they experienced any problems. The FIBRO-On program was created during the COVID-19 lockdown and since then, it has been successfully applied at the Central Sensitivity Syndromes Specialized Unit from the HUVH (Serrat et al., 2021a; Serrat et al., 2022b). See Tables 1, 2 for more details.

**TABLE 1:** Outline of the FIBROWALK program (with FIBRO-Out and FIBRO-On specifications).

Educational and psychological contents in the FIBROWALK program
• Pain Neuroscience Education (PNE)
• Cognitive Behavioral Therapy (CBT)
• Mindfulness training (MT)

**Physical therapeutic exercises in the FIBROWALK program**
• Warm-up: activation and mobility exercises
• Therapeutic exercises: moderate aerobic-cardiovascular and muscle strengthening exercises combined with some balance and coordination exercises performed in a playful manner with cognitive and emotional targets (multitask works) where the level of difficulty and dedication time gradually increases
• Cooling-down: flexibility and relaxation exercises

**Homework**
• Moderate intensity walking is recommended from the first session in a progressive pattern: 1st month - once per week, 2 nd month - twice per week, 3rd month - three times per week
• Exercises based on PNE, CBT and MT contents are also proposed every week (e.g., identifying danger signals, modifying sleep habits, challenging negative automatic thoughts, doing a 10-min body scan meditation)
• Double tasks: combining TE with CBT by fostering different cognitive/affective regulation skills throughout exercise (e.g., identifying external and internal stimuli which may promote positive affect during the exercise, discovering automatic thoughts during the exercise and uncovering patterns between thoughts with negative emotions, pain experience and fatigue; diminishing catastrophic thinking; recognizing the achievements, improving self-efficacy)

**Contents in each session#**
Session 1: PNE (1.2) + CBT (1) + MT (1)
Session 2: PNE (3.4) + CBT (2) + MT (2)
Session 3: PNE (5.6) + CBT (3) + MT (3)
Session 4: PNE (7.8) + CBT (4) + MT (4)
Session 5: PNE (9.10) + CBT (5) + MT (5)
Session 6: PNE (11) + CBT (6) + MT (6)
Session 7: PNE (12) + CBT (7.8) + MT (7.8)
Session 8: PNE (13) + CBT (9) + MT (9)
Session 9: PNE (14) + CBT (10) + MT (10)
Session 10: Family Session (PNE 1–16)
Session 11: PNE (15) + CBT (11) + MT (11)
Session 12: PNE (16) + CBT (12) + MT (12)

**Overall structure of the sessions for the FIBRO-Out and FIBRO-On versions of the FIBROWALK program**
**FIBRO-Out program (face-to-face sessions)**
• Review Phase (15′): Brief review of contents of the previous session
• Educational and psychological contents and Physical therapeutic exercises (1h 40′)
• Homework explanation (5′)

**FIBRO-On program (online videos)**
• Review Phase (2′): Brief review of contents of the previous session
• Educational and psychological contents and Physical therapeutic exercises (55′)
• Homework explanation (3′)
Handouts and specific practices are facilitated in each video session and, although the FIBRO-On videos’ duration is of 60 min each, the recommendation is to expend 120 min to do all the exercises proposed

**Note:** Both versions of the FIBROWALK (i.e., FIBRO-Out and FIBRO-On) are equivalent regarding contents and distribution of them across sessions. **#**The numbers between brackets correspond to specific contents of the program explained on Table 2. PNE , pain neuroscience education; CBT , cognitive behavioral therapy; TE , therapeutic exercise; MT , mindfulness training.

**TABLE 2:** Contents of the FIBROWALK program (i.e., Pain Neuroscience Education, Therapeutic Exercise, Cognitive Behavioral Therapy, and Mindfulness Training).

Pain neuroscience education (PNE)
1. Disassembling beliefs
2. Danger signals: modulation and modification
3. Concept of pain, fatigue, and pain system
4. Concept of central nervous system and central sensitization. The role of the brain
5. Acute vs. chronic pain: The purpose of acute pain and how it originates in the nervous central system
6. Pain vs. damage
7. Pain neuromatrix theory and representation of the virtual body
8. Nociception, nociceptors, action potential, peripheral sensitization, and synapses
9. Ascending and descending inhibitory pathways, spinal cord
10. Relationship with attention, perception, pain cognitions, and pain behaviors
11. Allodynia and hyperalgesia, hypersensitivity of the nervous central system
12. Pain memory, pain perception, and autoimmune evaluation error
13. Relationship with stress. Etiology
14. Neuroplasticity and how the pain becomes chronic
15. Relationship with emotions
16. Re-education, gradual activity, and therapeutic exercise
**Therapeutic Exercise (TE)**
1. Essential and necessary movement
2. Set a basal minimum of activity
3. Individualized gradual program
4. Small increases, patterns
5. Activities contingent on the task, not over time
6. Involvement in the tasks of daily life
7. Lifestyle change: cognitive coping strategies
8. Double tasks: focused on the following cognitive/affective targets: positive affect, negative affect, self-efficacy, catastrophic thinking, pain and fatigue
**Cognitive Behavioral Therapy (CBT)**
1. Relaxation and breathing
2. Modulating factors of pain
3. Catastrophizing and fear of movement (fear avoidance model)
4. Painful experiences: confrontation (fear avoidance model)
5. Vital values and setting goals
6. Organization of time
7. Sleep patterns
8. Sexual issues
9. Handling of attention
10. Cognitive restructuring
11. Emotional regulation tasks focused on identifying and promoting a more adaptive use of the following cognitive/affective regulation skills: self-blame, acceptance, rumination, focus on the positive, focus on plans, positive reassessment, put in perspective, catastrophizing, blaming others
12. Troubleshooting
**Mindfulness Training (MT)**
1. An Introduction to Body Scanning
2. Elementary Awareness
3. Sitting Practice and introduction to Yoga
4. Mindfulness and the brain
5. Mindfulness and communication: guilt, empathy, and conflict management
6. Responding vs. reacting
7. Dig deeper into personal practice
8. Mindfulness and Compassion: Strength vs. Cooperation
9. Stress Management
10. Thoughts Management
11. Management of difficult emotions or feelings
12. Dig deeper into personal practice

**Note:** More details on the interventions and program-associated materials and clinical guidelines (Serrat et al., 2022b) can be accessed through a reasonable request to the corresponding author.

#### 2.6.3 FIBRO-Out

The FIBRO-Out program includes the implementation of the 12-session FIBROWALK multicomponent program (Serrat et al., 2020) in an outdoor environment as an add-on to TAU. It will be conducted weekly and in group format (18–20 participants/group) and it will be carried out in a green area in an urban environment (i.e., “Parc del Cargol”) located in the neighborhood of the HUVH (Barcelona). FIBROWALK includes components of PNE, CBT, mindfulness training and therapeutic exercise. PNE aims to reconceptualize pain and is included in all sessions of the program. This is an educational program that teaches pain patients the difference between harm and pain, how the pain program is activated when actual or potential harm is appraised by the brain that seeks to generate an active protective response and how this assessment can be erroneous depending on different contextual factors. It is based on the “Explaining Pain” program by Butler and Moseley (2010). Examples, images and metaphors are used to facilitate the understanding of the educational content of the program (Nijs et al., 2011). Multi-component physical exercises such as stretching, balance training, posture correction, limb extension and low-impact walking (i.e., Nordic walking) are performed with a moderate training load. Exercises are individualized, establishing a baseline of minimum performance and guiding progression throughout the 12-week program. Following the recommendations of the *American College of Sports Medicine* (Pescatello et al., 2014), each therapeutic physical exercise session is structured in three parts: warm-up, main exercise and cool-down. At the beginning of each session, an interval of approximately 15 min is set aside to discuss the most important aspects of the task between sessions, as well as to review the concepts already explained in the previous treatment sessions. The psychological approach in the FIBROWALK program is based on CBT and aims to promote cognitive changes (e.g., reduce catastrophizing about pain), improve emotional regulation, increase sleep quality, enhance problem-solving strategies and facilitate the establishment of life values and goals (Moix and Kovacs, 2009). Mindfulness exercises are also performed with the aim of training attention to present experience and fostering a non-evaluative attitude towards that experience (Kabat-Zinn, 2013). Mindfulness exercises are based on the Mindfulness-Based Stress Reduction (MBSR) program (Kabat-Zinn, 2013), for which evidence of efficacy and cost-effectiveness in FM patients has been demonstrated (Pérez-Aranda et al., 2019). CBT and mindfulness training help patients to become aware of their own thoughts, emotions and behaviors so that, once identified, they can be regulated or managed facilitating the same situation (e.g., pain) to be experienced in a more flexible and fulfilling way, with less associated suffering. Consistent with PNE, directing attention to the present (rather than to future concerns or past losses or threats) may provide the appropriate safety setting to reduce the perception of alarm or danger associated with pain and allow for successful re-education of the pain program (Moseley G. L., 2003; Moseley L., 2003; Moseley et al., 2015). The framework of motivational interviewing (Nijs et al., 2020) and the cognitive-behavioral model of fear avoidance (Vlaeyen et al., 1995) are part of the theoretical background of CBT in the FIBROWALK program. The intervention is carried out by encouraging social interactions, supported by *role-playing* techniques to promote understanding of content and enhance adherence to treatment. In order to promote an adequate motivational state for the performance of all the exercises and practices in the FIBROWALK program, individualized modifications will be proposed following the trans-theoretical model of stages of change (Prochaska, 2008). For a summary of the contents of the FIBROWALK program, see Tables 1, 2.

In RCTs of non-pharmacological treatments, it is recommended that more than one therapist deliver each treatment to create a more realistic and generalizable impression of effectiveness (Ost, 2014). FIBRO-Out intervention will be conducted by six different therapists (i.e., three health psychologists and three physiotherapists) with experience in the field of chronic pain and FM. Psychotherapists and physiotherapists will work in pairs in each study group, leading psychotherapeutic or physiotherapeutic FIBROWALK components. The physiotherapist in charge of the group will conduct the first block of each session of the program (PNE + therapeutic exercise; 1 h duration) and the psychologist will conduct the second block (CBT + mindfulness; 1 h duration). Four different pairs of therapists will be generated to lead the four FIBRO-Out groups (18–20 participants per group) that will be carried out for this study. The outdoor FIBROWALK program has already been the subject of a previous investigation by the group (Serrat et al., 2020) and very high levels of adherence were observed, with 90% of patients attending 10 or more sessions out of 12.

### 2.7 Treatment fidelity evaluation of the FIBRO-Out arm

All therapists involved in this treatment arm will have prior experience in CBT or physiotherapy applied to individuals with FM or chronic pain and will receive an 8-h face-to-face training prior to starting the RCT with the aim of standardization. The six therapists in the FIBRO-Out program will be trained by fulfilling the FIBRO-On program (which includes all the same components as the outdoor format) and participating as co-therapists in a brief FIBRO-Out program (i.e., 4 sessions, 2 h each) for people with FM with the close face-to-face supervision of the clinical manager of the study (MS). All program-related doubts raised in each training session will be shared within the therapist group and solved by MS. Furthermore, every week during the intervention, meetings will be held between the therapists and MS to allow them to discuss any difficulties experienced and find ways to overcome them. To allow quality control of the provided treatment, the training of the therapists will be based on the FIBROWALK manual and guidelines (Serrat et al., 2021a; Serrat et al., 2021b; Serrat, 2022a) and the therapists will be evaluated for treatment fidelity for independent experts at the end of their intervention. This program manual will be used for training, supervision of the therapists and also for monitoring the program’s quality and performance. To monitor treatment fidelity within the FIBRO-Out arm, research assistants will audio-tape all treatment sessions and two independent experts in the program will rate adherence to each component of the FIBRO-Out. A random sample of tapes, stratified by therapist and therapy session will be rated using the instruments described below. Treatment fidelity will be assessed with a measure of fidelity criteria based on the guidelines for the FIBROWALK intervention, which will be generated by MS before the start of the interventions. The results of the evaluation of the therapist’s adherence to the program will not only allow monitoring of the program’s quality but also allow providing detailed feedback to the therapists about their performance.

All study participants will be asked to continue taking the same medication regimen for the duration of the study period (6 months).

### 2.8 Study measures

Assessment of outcome and process measures will be carried out at baseline, 6 weeks after starting treatment, at post-intervention (12 weeks) and at 6-month follow-up after starting the trial (follow-up. Cost-utility assessment will be conducted at baseline and at the follow-up and biomarkers evaluation at baseline and post-intervention. The research assistants in charge of the evaluations will be extensively trained by researchers (MS, AF-S), who have broad experience in applying the study measures.

The following domains will be assessed (see Table 3 for a summary of assessments):

- Socio-demographic questionnaire: gender, date of birth, marital status, cohabitation, educational level and employment status.
- Clinical data: date of FM diagnosis, years with a FM diagnosis, comorbidity with other diagnosed medical/psychiatric conditions, type and dosage of current medication, weight and height.

**TABLE 3:** Summary of the assessments in the RCT.

	Baseline	6 weeks	Post-intervention (12 weeks)	Follow-up (6 months)
Sociodemographic and clinical characteristics of the sample				
Sociodemographic data	X			
Clinical data	**X**			
Primary outcome measure				
FIQR	**X**	**X**	**X**	**X**
Secondary outcome measures				
VAS Pain	**X**	**X**	**X**	**X**
VAS Fatigue	**X**	**X**	**X**	**X**
HADS	**X**	**X**	**X**	**X**
PSS	**X**	**X**	**X**	**X**
PF-SF-36	**X**	**X**	**X**	**X**
B-PSQI	**X**	**X**	**X**	**X**
MISCI	**X**	**X**	**X**	**X**
CSI	**X**	**X**	**X**	**X**
PGIC/PSIC			**X**	
Process measures				
PCS	**X**	**X**	**X**	**X**
TSK-11	**X**	**X**	**X**	**X**
PIPS	**X**	**X**	**X**	**X**
R–NPQ	**X**	**X**	**X**	**X**
Cost-utility measures				
CSRI	**X**			**X**
EQ-5D	**X**			**X**
Other self-reported measures				
CEQ	**X**		**X**	
Perceived adverse effects of the interventions		**X**	**X**	
Fidelity evaluation (adherence of the therapists to the FIBROWALK guidelines)			**X**	
physiological markers				
Immune-inflammatory markers	**X**		**X**	
BDNF	**X**		**X**	

Note: BDNF, Brain-Derived Neutrophic Factor; B-PSQI, brief version of the pittsburgh sleep quality index; CEQ, Credibility/Expectancy Questionnaire; CSI, central sensitization inventory, short form; CSRI, client service receipt inventory; HADS, hamilton anxiety depression scale; MISCI, multidimensional inventory of subjective cognitive impairment; PCS, Pain Catastrophizing Scale; PF-SF-36, Physical Function from the Short Form-36 Health Survey; PGIC/PSIC, Patient Global Impression of Change/Pain Specific Impression of Change; PIPS , psychological inflexibility in pain scale; PSS, perceived stress scale; R-NPQ, revised neurophysiology of pain questionnaire; TSK, tampa scale for kinesiophobia; VAS, Visual-analogue scale.

#### 2.8.1 Primary clinical outcome

The *Revised Fibromyalgia Impact Questionnaire* (FIQR; Luciano et al., 2013) is a 21-item questionnaire (0–10 scale) which assesses the dimensions of physical dysfunction, overall impact of FM and severity of the symptoms (i.e., pain, energy, stiffness, sleep quality, depression, memory issues, anxiety, pain to the touch, balance problems, and increased sensitivity to noises, lights, smells, or temperatures), and is used to measure the impact of FM over the past week. This questionnaire is currently considered the “gold standard” for assessing functional impairment in patients with FM. A total score for FIQR ranging from 0–100 can be obtained by adding the 3 subscales, with higher scores indicating greater FM severity. The Spanish version of the FIQR has an excellent internal consistency (α = 0.91–0.95) (Luciano et al., 2013).

#### 2.8.2 Secondary clinical measures

- *Visual-analogue scale of perceived pain* (VAS-Pain; Serrano-Atero et al., 2002) in which patients indicate their pain during the last week on a 10 cm line (0 = No pain, 10 = Unbearable pain).

- *Visual-analogue scale of perceived energy/fatigue* (VAS-Fatigue; Serrano-Atero et al., 2002) in which patients indicate their fatigue during the last week on a 10 cm line (0 = Lots of energy, 10 = No energy).

- *Hospital Anxiety and Depression Scale* (HADS; Luciano et al., 2014a). HADS is used to quantify the severity of anxiety and depression symptoms. It consists of two dimensions (anxiety and depression) of 7 items each responding on a Likert scale of 4 points. Total scores of each scale (HADS-A and HADS-D) range from 0 to 21, where higher scores indicate greater symptom severity. The Spanish version of the HADS has demonstrated satisfactory internal consistency for anxiety (α = 0.83) and depression (α = 0.87) subscales inpatients with FM (Luciano et al., 2014b).

- The *Perceived Stress Scale*, short form (PSS; Herrero and Meneses, 2006) is a 4-item scale used to evaluate the degree to which respondents appraise situations as stressful in the last month with responses scored on a Likert scale between 0 = “never” and 4 = “very often,” and with total scores ranging from 0 to 16 for the short 4-item version of the scale. Higher scores indicate greater perceived stress. The Spanish version of the 4-item version of the PSS shows acceptable internal consistency (Cronbach’s α = 0.77).

- The *physical function subscale of the Short Form-36 Health Survey* (PF-SF-36; Alonso et al., 1995) is used to measure physical function. This dimension comprises a total of 10 items, which are answered on a Likert scale of 3 points. Total scores on each scale are transformed and can range from 0 to 100, with higher scores indicating better physical function. The Spanish version of the PF-SF-36 shows adequate internal consistency (α = 0.94).

- *Brief version of the Pittsburgh Sleep Quality Index* (B-PSQI; Sancho-Domingo et al., 2021). The PSQI is the most widely used questionnaire in research and clinical practice to assess sleep quality. The B-PSQI is a brief version of this measure including six questions about sleep quality. The Spanish version of the B-PSQI shows adequate internal consistency (α = 0.79).

- The *Multidimensional Inventory of Subjective Cognitive Impairment* (MISCI; Feliu-Soler et al., 2018) is a 10-item scale with a 5-point Likert scale (from 1 “Not at all/Never” to 5 “Very much/Always”) for assessing perceived cognitive dysfunction in FM during last week. It includes the cognitive domains: mental clarity, memory, attention/concentration, executive functioning, and language. The MISCI was developed through classical test theory and item response theory from cognitive functioning item banks that were developed as part of the Patient Reported Outcomes Measurement Information System (PROMIS). The Spanish version of the MISCI shows excellent internal reliability (α = 0.91).

- The *Central Sensitization Inventory, short form* (CSI; Nishigami et al., 2018) is the brief form of the CSI (Cuesta-Vargas et al., 2016), and consists of two parts: part A includes nine items scored from 0 to 4, with higher total scores reflecting increased central sensitization symptoms; part B (which is not scored) determines whether one or more specific disorders of central sensitization related to pathophysiology (i.e., restless legs syndrome, chronic fatigue syndrome, FM, temporomandibular joint disorder, migraine or tension headaches, irritable bowel syndrome, multiple chemical sensitivities, neck injury, anxiety or panic attacks, and depression) have been diagnosed before. Adequate internal consistency for the CSI-9 has been also reported (Cronbach’s alpha = 0.80).

#### 2.8.3 Cost-utility measures

- *Client Service Receipt Inventory* (CSRI; Vázquez-Barquero et al., 1997). The CSRI is a questionnaire for economic evaluation. The version used in this study is designed to retrospectively collect data on the use of health and social services during the previous 6 months.

- EuroQoL-5D-5L (EQ-5D-5L; Badia et al., 1999). The EQ-5D-5L is an instrument for assessing health-related quality of life. It consists of two parts: the first part assesses the individual’s difficulties in relation to mobility, self-care, pain/discomfort and anxiety/depression; and the second part assesses the patient’s current perceived health status on an analogue-visual scale from 0 (“the worst health you can imagine”) to 100 (“the best health you can imagine”).

#### 2.8.4. Process measures

- *Pain Catastrophizing Scale* (PCS; García-Campayo et al., 2008). The PCS is used to evaluate catastrophic thoughts associated with pain. It consists of three dimensions (e.g., rumination, magnification, and helplessness) with 13 items in total, which are answered on a Likert scale of 5 points. Total scores on each scale range from 0 to 52, with higher scores indicating more catastrophic thoughts. The Spanish version of the PCS shows adequate internal consistency (α = 0.79).

- *Psychological Inflexibility in Pain Scale* (PIPS; Wicksell et al., 2008). The PIPS is a 12-item scale that assesses psychological inflexibility in pain patients, and includes two factors: avoidance and cognitive fusion with pain. The items consist of different statements that are considered to be related to chronic pain, psychological inflexibility, suffering and disability (coherent with the ACT theory). All the items are rated on a 7-point Likert scale that ranges from “1 = never true” to “7 = always true”, with higher scores indicating more psychological inflexibility. The Spanish version of the PIPS (Rodero et al., 2013) shows adequate internal consistency (α = 0.90).

- *Tampa Scale for Kinesiophobia* (TSK; Gómez-Pérez et al., 2011). The TSK is a measure aimed at assessing fear of movement and comprises 11 items in a 4-point Likert scale with a total score ranging from 11 to 44, with higher scores indicating greater pain and fear of movement. The Spanish version of the TSK has an adequate internal consistency (α = 0.79).

- The *Revised Neurophysiology of Pain Questionnaire* (R-NPQ; Torres-Lacomba et al., 2021). The R-NPQ consists of 13 statements (True/False/I do not know) about pain neurophysiology and has been extensively used to assess pain biology knowledge. The R-NPQ score ranges from 0 to 13 (sum of all correct items), and can be also expressed as a rate of correctly answered items. Excellent internal consistency for the R-NPQ has been reported in Spanish samples (α = 0.87–0.92).

### 2.9 Other self-reported measures

- The *Patient Global Impression of Change (PGIC)* and the *Pain Specific Impression of Change* (PSIC) (Scott and McCracken, 2015) are measures to assess, respectively, self-perceived meaningful clinical change (on a 7-point Likert scale, from 1 = “Much better” to 7 = “Much worse”) in general or in the following specific domains: physical and social functioning, work-related activities, mood, and pain.

- Adapted version of Credibility/Expectancy Questionnaire (CEQ; Devilly and Borkovec, 2000). It is a 6-item questionnaire widely used to assess treatment expectancy as well as the credibility of the FIBRO-On and FIBRO-Out interventions. The first part of the CEQ comprises three items focused on therapy credibility (i.e., the extent to which the treatment appears a)logical and b) useful, and c) the confidence with which the treatment’s patient would recommend this one to a friend with the same health condition); and three more items evaluating expectations (i.e., the extent to which the patient thinks an improvement will happen as a consequence of the treatment; the extent to which the patient really feels that the intervention will reduce him/her symptoms, and the extent to which the patient really feels a symptom improvement will occur). After finishing the treatment, the patient’s opinion regarding the treatment received will be also gathered by using the second part of the CEQ which includes the same questions as in the first part of the CEQ but in past tense. The CEQ showed overall adequate internal consistency (α = 0.84–0.85) in a previous study in a similar clinical sample (Pérez-Aranda et al., 2019).

- An *ad hoc* item (i.e., *Have you experienced, during the course of the treatment, any unwanted symptom that you think might be directly or indirectly associated with the intervention?*) to check for the presence of adverse effects (e.g., headaches, dizziness, sleep problems, *etc.*) associated with the evaluated interventions. This measure has been used in a previous study (Pérez-Aranda et al., 2019) and will be administered both at 6-week and post-intervention assessments.

- Patients’ adherence evaluation to the FIBRO-On and FIBRO-Out programs. An *ad hoc* instrument (i.e., personal practice log) for the weekly recording of home practice will be developed to determine level of adherence to psychotherapeutic and therapeutic exercise recommended homework.

### 2.10 Physiological markers

Blood samples will be collected in vials which will be centrifuged and the resulting serum will be stored frozen at -80°C until analysis. All samples (pre and post) will be analyzed in a single analytical batch in order to reduce inter-assay variability (approximately 15%). Serum levels of the cytokines IL-6, CXCL8, IL-4, IL-10, IL-17A and hs-CRP will be assessed. For the quantification of cytokines, Milliplex^®^ reagents from MerckMillipore analyzed on a Luminex^®^ platform will be used. The highly sensitive Human High Sensitivity T Cell multiplex kit will be used (catalogue number: HSTCMAG28SPMX11). The hs-CRP will be quantified by turbidimetry on a Siemens Atellica autoanalyzer. BDNF levels will be evaluated using an ELISA kit (reference SEA011Hu-96T). Sample analysis will be performed by the Echevarne Laboratory. Detection concentration ranges: IL-6 (0.64–10,000 pg/ml), CXCL8 (0.64–10,000 pg/ml), IL-4 (0.64–10,000 pg/ml), IL-10 (2.6–40,000 pg/ml), IL-17A (1.3–20,000 pg/ml), hs-CRP (0.1–50 mg/L), and BDNF (31.2–2,000 pg/ml). Biomarkers will be assessed only at baseline and post-intervention because of the following reasons: a) evidence of changes in immune-inflammatory markers and BDNF levels after non-pharmacological interventions with a similar duration (Sanada et al., 2015; Montero-Marín et al., 2019; Pérez-Aranda et al., 2019); b) lower risk of sample loss (vs. assessing at 6 months); c) possibility to use pre-post change as a mediator of clinical changes at 6 months follow-up; and d) budget constraints.

### 2.11 Statistical analyses

SPSS v26.0, STATA v16.0 and Mplus v7.4 will be used for the statistical analyses. Descriptive statistics will be calculated for all variables and presented as means and standard deviations if continuous, or as absolute numbers and percentages (%) if categorical. The Levene test will be used to assess the equality of variances of continuous variables and Kolmogorov–Smirnov to test sample normality and distribution. One-way ANOVAs (with post hoc Tukey’s HSD or Games-Howell tests) for continuous values and χ2 tests with continuity corrections for categorical values will be computed on all baseline measures and socio-demographic variables to examine between-group differences at baseline.

#### 2.11.1 Analysis of clinical effectiveness

The primary effectiveness analysis will be conducted on an intention-to-treat (ITT) basis with the FIQR total score as a primary clinical endpoint (McCoy, 2017) and by linear mixed-effects regressions with restricted maximum likelihood (REML). REML accounts for the correlation between repeated measures for each individual and produces less biased estimates of variance parameters when using small sample sizes or unbalanced data (Egbewale et al., 2014). No imputation of missing data will be conducted, since it has been reported that multiple imputation is not necessary for all types of missing data (missing completely at random, missing at random, and missing not at random) before computing longitudinal mixed model analysis (Twisk et al., 2013). Unstandardized regression coefficients (B) and 95% confidence intervals (95% CIs) will be computed for the ‘group x time’ interaction between groups at 6-week, post-treatment and 6-month follow-up assessments. Cohen’s d will be calculated for each pairwise comparison, using the pooled baseline SD to weight the differences in the pre–post mean values and to correct for the population estimate (Morris, 2008). The rule of thumb for effect size interpretation will be the classical cutoffs for Cohen’s d of 0.20 = small, 0.50 = medium, and 0.80 = large. All secondary clinical endpoints will be analyzed using the same statistical procedure. Benjamini–Hochberg correction for multiple comparisons will be applied to control for false rate discovery (Glickman et al., 2014). All the analyses will be replicated using a “completers” approach including all participants who finished the study, and a per-protocol approach, taking into account only those patients who attended at least 75% of the sessions (9 out of 12).

In addition, to assess clinical significance of the improvement on the primary outcome (FIQR), all the participants will be allocated into the categories of “responder” and “non-responder” by using the criteria of presenting at least a 20% reduction in the pre–post FIQR total score or not, respectively (Bennett et al., 2009). This classification will be used to compute the number needed to treat (NNT) for each treatment when compared to the others. The NNT refers to the estimated number of patients who need to be treated with a new proposed treatment (i.e., rather than the control comparison treatment) for one additional patient to benefit. A 95% CI for each NNT will be calculated. This index allows findings from RCTs to be more meaningful to clinicians. Furthermore, in order to explore potential predictors of treatment response in each intervention arm, t-tests and χ2-tests will also be performed to assess potential baseline differences in sociodemographic, clinical and physiological variables between responders vs. non-responders within the FIBRO-Out and FIBRO-On arms. Finally, baseline differences between participants who completed all study assessments and those who did not will also be evaluated (with *t* and *χ2*-tests) to detect any potential attrition bias.

The differences between FIBRO-On and FIBRO-Out on perceived clinical improvement (assessed with PGIC and PSIC) and on treatment expectation/opinion (i.e., with the CEQ) will be computed using χ^2^ tests and Student’s *t* tests, respectively.

#### 2.11.2 Mediation analyses

We will compute pre–post change scores for all process measures in the study and pre–follow-up change scores for primary and secondary outcomes. Bivariate Pearson correlations will be calculated between the pre–post change scores for the process variables and the pre–follow-up change scores for the clinical endpoints to detect statistically significant relationships. The direct and indirect associations between the treatment condition (i.e., FIBRO-On vs. TAU, FIBRO-Out vs. TAU, or FIBRO-On vs. FIBRO-Out, as independent variable), process variables (mediators), and primary and secondary outcomes (dependent variables) will be computed by using path analyses. With this statistical approach, we are considering temporality into account, which increases the prospect of establishing conclusions about causality. Simple and multiple mediation (simultaneously testing multiple variables as mediators) models will be computed.

Similarly, for obtaining more detail about psychological mechanisms fostered during intervention and its role in the observed effect at post-intervention, mediation analyses will also be conducted by using change scores of process measures between baseline and 6-week assessment and change scores for clinical measures between baseline and post-intervention assessments. The direct paths between treatment condition and clinical outcomes and the indirect effect path through the mediation variables will be tested in all the models. Regression coefficients (B) of bias-corrected bootstrapped indirect effects will be calculated as well as their SEs and 95% CIs (Lockhart et al., 2011). Parameters of indirect effects are considered statistically significant when the 95% CI did not include 0. Participants with missing data will be excluded for this analysis.

#### 2.11.3 Cost-utility analysis

The economic evaluation will be reported according to the Consolidated Health Economic Evaluation Reporting Standards statement (Husereau et al., 2013) and the Good Research Practices for Cost-Effectiveness Analysis Alongside Clinical Trials (Ramsey et al., 2015). The approach we will follow is estimating costs from the healthcare and the government perspectives, taking the previous 6 months as the time frame. Catalonia has full governance of health and social care and, as in every other Spanish region, healthcare is universal and publicly financed. The government perspective includes both direct healthcare costs assumed by the Catalan government (excluding out-of-pocket costs and costs associated with private insurance) and indirect costs related to productivity losses assumed by the Spanish government. The healthcare perspective includes only direct healthcare costs. Costs will be estimated for the 6 months before starting the intervention (at baseline assessment) and for the 6 months before the follow-up assessment.

Direct costs will be calculated as the sum of medication costs and health service use (primary, specialized and emergency care consultations, hospital admissions) and cost of staff to run the interventions. The cost of medication will be calculated by determining the price per milligram during the study according to the Vademecum International (Red Book), and including VAT. Total medication costs will be computed by multiplying the price per milligram by the daily dose in milligrams and the number of days receiving the specified treatment. The unit costs of medical tests and health services will be obtained from the SOIKOS health database (http://www.oblikue.com/bddcostes/). Indirect costs (lost productivity) will be computed according to the human capital approach, by multiplying the minimum daily wage in Spain in 2022 by the number of days on sick leave reported by each participant. Unit costs will be reported in Euros (€).

The utilities represent the rating of the patients’ quality of life on a scale from 0 (“as bad as death”) to 1 (“perfect health”). For the cost-utility analysis, the ratio between the cost of each intervention and its consequences in terms of QALYs will be calculated. QALYs will be computed using Spanish EQ-5D tariffs. Following the International Society for Pharmacoeconomics and Outcomes Research (ISPOR) core recommendations for cost-effectiveness analyses alongside RCTs (Ramsey et al., 2015), we will calculate the incremental cost-utility ratios (ICUR) for each pairwise comparison (TAU vs. FIBRO-On, TAU vs. FIBRO-Out, FIBRO-On vs. FIBRO-Out), defined as the difference in mean costs divided by the difference in mean QALYs. Given that the duration of the study is 6 months, neither costs nor outcomes are subject to discounting. QALYs at 6 months after the start of the interventions will be approximated by calculating the area under the curve.

Cost-effectiveness analyses will be performed using the Zellner’s seemingly unrelated regression (SUR) model (Greene, 2003) with the STATA’s *sureg* command. Using the SUR method for cost-effectiveness purposes implies the use of a bivariate system of regressions that includes both costs and outcomes (with the latter being either QALYs or EurQol-5D-5L VAS, depending on the model considered) as the dependent variables of the two separate equations, which will be estimated jointly. The regressions of costs and outcomes are therefore part of two regressions on treatment allocation (i.e., whether they are assigned to TAU, FIBRO-Out, or FIBRO-On) plus an additional set of control variables (measured at baseline) if necessary: age, gender, marital status, education level, employment status, baseline costs, or baseline outcome, depending on the equation considered.

Estimates of incremental cost and of incremental effect values using the SUR method described above will be derived with 1,000 bootstrap replications in order to address a possible skewness in the distribution of the dependent variables (Briggs et al., 1997). We also will construct acceptability curves to represent the probability of the intervention being cost-effective, given a varying threshold for the willingness to pay for each QALY gained in each intervention compared to the others. The robustness of the cost-utility results will also be tested in different case scenarios; more precisely, we will use ITT approach as the primary analysis, imputing missing values for those variables missing at 6-month follow-up by using multiple imputation methods with the chained equations approach (Royston and White, 2015). Additional scenarios (sensitivity analysis) will be also conducted repeating the same statistical analyses in a complete case analysis, including only those participants with complete assessments at baseline and at 6-month follow-up, and in a per-protocol analysis including only those participants who attended at least 75% of the sessions (9 out of 12). STATA 16.0 statistical software will be used for cost-utility analyses.

#### 2.11.4 Analysis of changes in biomarkers

The effect of interventions on immune-inflammatory markers and BDNF levels will be evaluated also with REML. In those cases where cytokine concentrations are under detection level of the test, the detection limit value will be assigned. Cytokines, hs-CRP and BDNF values will be subjected to a natural logarithmic transformation to normalize the significantly skewed data distributions. In order to comprehensively integrate pro-inflammatory with anti-inflammatory markers, indexes indicative of inflammatory balance will be calculated (e.g., IL-6/IL-10, CXCL8/IL-10 and hs-CRP/IL-10) in a similar way to previous studies (Andrés-Rodríguez et al., 2019). Since some pharmacological treatments such as antidepressants are reported to potentially affect some cytokine levels (Hannestad et al., 2011), differences in such levels between patients taking vs. not taking these drugs will be also evaluated. In case of any statistically significant effect, antidepressant status (“taking” = 1, “not taking” = 0) will also be included as a co-variable in the REML analyses for each specific biomarker.

### 2.12 Ethical issues of the project

Written informed consent will be obtained from all participants prior to randomization. Participants will be provided with a general overview of the aims and characteristics of the study and the interventions before signing the informed consent. They will also be assured that they will be participating voluntarily and can withdraw at any time from the study with the guarantee that they will continue to receive the most appropriate medical treatment prescribed by their general practitioner. The study will be conducted in compliance with the Declaration of Helsinki (version in force; currently Fortaleza, Brazil, October 2013). The study will be conducted in accordance with the protocol and with the relevant legal requirements: Law 14/2007 of July 3, on Biomedical Research. The VHIR Research Committee Board evaluated and approved the study protocol in May 2022 [PR (AG)99/2022]. All patient data will be treated as confidential and only the research team will be allowed to access it after recodification of name and personal identity number (so no individual can be directly identified). Only the principal investigator (AF-S) and the clinician coordinator of the study (MS) will have access to the code key which will be stored separately in a safe place in accordance with Spanish legislation. All data will be computer processed and stored. Blood samples will be stored encrypted, and will not be directly traceable back to individuals. Blood samples will be used only in ways to which the participants consented and may only be made available to a new research project after Ethical Research Committee approval and participants provide a new agreement. Furthermore, the participants have the right to request, without explanation, that their samples be destroyed or made completely anonymous.

### 2.13 Blinding

Randomization and group allocation will be completely masked for the study assessors and study participants will be asked not to communicate with the assessor about the treatment received. The study participants will be provided with a summary of evidence of the FIBROWALK program which constitute the basis for FIBRO-On and FIBRO-Out programs. As it is usual in non-pharmacological trials, neither participants nor the therapists can be blinded to treatment allocation. The therapists in the FIBRO-On and FIBRO-Out arms will not be involved in any assessment task of the study.

### 2.14 Forecast execution dates

Initial recruitment of patients: September 2022.

Finalization of patient recruitment: January 2023.

Finalization of patient monitoring period: July 2023.

Publication of results: December 2023.

## 3 Discussion

The On&Out study will evaluate the effectiveness, cost-utility and the physiological effects of adding the FIBROWALK program in Online (i.e., FIBRO-On) or in Outdoor format (i.e., FIBRO-Out) to TAU. In order to determine the mechanisms of action of these interventions, we will also evaluate potential process measures of these interventions, as well as physiological markers with evidence of alteration in FM and that are likely modifiable by non-pharmacological interventions. Three previous studies evidenced the short-term efficacy (i.e., post-intervention) compared to TAU of both the FIBRO-On (Serrat et al., 2021a; Serrat et al., 2022b) and FIBRO-Out programs (Serrat et al., 2020); however, the present RCT will evaluate for the first time if these clinical effects are maintained at 6-month follow-up along with the aforementioned analyses on cost-utility, mediation and physiological markers.

The fact that FIBROWALK’s tested interventions are in online format (FIBRO-On) or in group format (FIBRO-Out) can make them more cost-effective, and therefore of interest for health managers. Additionally, both formats can be considered “safe” in epidemiological terms (compared to face-to-face indoor classical interventions) as they do not imply in closed spaces shared with other people where exists a higher risk for contagion (e.g., Bazant and Bush, 2021). Furthermore, particularly for the FIBRO-On intervention, these therapies can also be followed under strict social distancing measures and even in lockdown circumstances. If the results of this RCT are strong enough in terms of efficacy and cost-utility, FIBRO-Out and/or FIBRO-On could be considered for their inclusion in the public healthcare Spanish system to treat individuals suffering from FM. Additionally, this RCT may also pave the way for further RCTs testing the efficacy of these interventions in other chronic diseases cursing with central sensitization (e.g., irritable bowel syndrome, chronic headache, temporomandibular disorder, pelvic pain syndromes), along with other emerging conditions such as persistent post-COVID syndrome which may display overlapped symptoms with FM (Haider et al., 2022).

The On&Out study will also evaluate the effect of the FIBROWALK multicomponent programs on immuno-inflammatory markers and BDNF levels that seem to be altered in FM and/or play a role in the effectiveness of non-pharmacological treatments (Rodriguez-Pintó et al., 2014; Andrés-Rodríguez et al., 2019; Montero-Marin et al., 2019). It should also be noted that, to date, the effect of any multicomponent program on such biomarkers in FM has not been evaluated by means of a RCT design. Furthermore, the evaluation of changes in these biomarkers and their association with clinical change will further deepen the understanding of the role of the immune system and neuroprotective agents in FM and related disorders cursing with central sensitization. Associated with this substudy of the project, it should be noted that the inclusion of biomarkers may also make it possible to determine different patient profiles (based on their psychobiological characteristics) which could be predictive of a greater or lesser response to the treatments under study. This aspect is fully connected with the emerging field of personalized treatments and can contribute to optimizing the use of healthcare services while also enhancing the efficacy and cost-effectiveness of the interventions evaluated (Thase, 2014; Lopresti, 2017; Carvalho et al., 2019).

This study has some strengths that should be highlighted. The inclusion of a relatively large sample and of a comprehensive set of measures will allow us to compare the short term (pre-post) and long-term (6 months) effectiveness of two different formats of the FIBROWALK multicomponent program on core FM outcomes and psychological process measures. Furthermore, the identification of cost-effective interventions is a priority for the national health system, especially for highly prevalent conditions such as FM. This study will evaluate for the first time the cost-effectiveness of two formats (which can be particularly useful in the pandemic and post-pandemic era) of a multicomponent intervention for FM. In this sense, it is necessary to introduce new practices in the provision of healthcare services to improve efficiency in the use of resources. In this regard, boosting treatments aimed at changing lifestyles and patterns of cognition and behavior (as it is aimed in FIBROWALK) constitutes a strategy of financial sustainability as, in the long-term, it will allow an extension of the culture of health understood holistically and, therefore, a decrease in the burden of disease on society as a whole. Our study will also explore immune-inflammatory and neuroprotective pathways behind the efficacy of the evaluated treatments, and will combine clinical and process measures which may lead to a better characterization of the syndrome, to new therapeutic interventions, and to a better prediction of treatment response in order to better determine what works for whom in the field of personalized medicine. Finally, it is also worth mentioning that the present RCT will involve six therapists (three psychologists and three physiotherapists) in delivering the FIBRO-Out intervention; doing so (instead of only one therapist delivering the therapy) can provide more realistic and generalizable results (Ost, 2014) which can be useful for the future implementation of these interventions in healthcare systems.

There are also some potential limitations that should also be recognized in this RCT. One of the main risks of this project may be dropouts and non-adherence, which are expected to be higher than in previous studies that only evaluated the short-term efficacy of the interventions (Serrat et al., 2020; Serrat et al., 2021a; Serrat et al., 2021b; Serrat et al., 2022b). In this regard, since previous studies showed very high adherence to the programs (over 90%; Serrat et al., 2020; Serrat et al., 2022b), a dramatically high dropout in the present study is not expected; however, to limit the effect of these dropouts and lack of adherence in our results, we will conduct several sensitivity analyses (ITT, completers, per protocol analysis). We also have to note the lack of blinding of participants and therapists, which is a typical bias in any RCT including non-pharmacological treatments. Although a weekly questionnaire will be administered to check the adherence to FIBRO-On program (i.e., watching the videos), there is a risk that some participants in the FIBRO-On group do not watch the videos and inform differently. It is also relevant to highlight that this RCT will be carried out in a specialized unit of tertiary referral hospital in the context of clinical practice. Related to this, stricter inclusion/exclusion criteria could not be established due to pressures in daily clinical practice (i.e., most patients will be admitted). At the same time, this latter point may have a positive aspect by increasing the external validity of the study and so the generalization of the results into the “real” clinical practice.

Up to date, there is no study assessing the cost-effectiveness of multicomponent approaches for FM neither evaluating the long-term effectiveness of such type of interventions in online or outdoor formats. If one or both treatments evaluated in this present RCT (i.e., FIBRO-On and/or FIBRO-Out) shows to be effective and cost-effective they should be included as part of the standard of care for treating FM. Furthermore, since both interventions are, per definition, conducted out of the hospital settings, they could also be safely administered in the context of future pandemics, and (in the particular case of the FIBRO-On program) in circumstances of low accessibility to effective treatments for FM (e.g., in remote areas), highly congested healthcare services and/or low availability of clinical resources. The On&Out project is also aimed at determining psychophysiological patient profiles which can be useful for identifying potential treatment beneficiaries (i.e., treatment responders) beforehand which may in turn improve healthcare resources allocation. Last but not least, the present study may also help us in deepening our knowledge on which psychological and physiological mechanisms underpin the clinical effects of non-pharmacological interventions in FM, further contributing to the potential detection of novel therapeutic targets.

## Data Availability

The original contributions presented in the study are included in the article/supplementary material, further inquiries can be directed to the corresponding author.
